# Structural Vulnerability and Police Interaction among Women Who Use Drugs amid De Facto Decriminalization in Baltimore, Maryland

**DOI:** 10.1007/s11524-025-01030-6

**Published:** 2026-02-24

**Authors:** Laura Nicole Sisson, Saba Rouhani, Catherine Tomko, Natalie Flath, Susan G. Sherman

**Affiliations:** 1 Department of Health, Behavior, & Society, Johns Hopkins Bloomberg School of Public Health, 1812 Ashland St, Baltimore, MD USA; 2 Department of Mental Health, Johns Hopkins Bloomberg School of Public Health, Baltimore, MD USA; 3 Department of Epidemiology, New York University School of Global Public Health, New York, NY USA

**Keywords:** Policing, Drug use, Decriminalization, Structural vulnerability

## Abstract

Exposure to criminal-legal systems, including policing, arrest, and incarceration, has deleterious effects on access to health and social services among people who use drugs. Women who use drugs (WWUD) may be especially vulnerable to policing, due to the high prevalence of sex work among them, which is also a criminalized behavior. Recent epidemiologic events and policy reforms are thought to have reduced exposure to arrests for low-level, non-violent crimes; in Baltimore City, this decline was demonstrated following the COVID-19 pandemic and implementation of de facto decriminalization of misdemeanor offenses including drug possession and solicitation (prostitution). However, possible impacts of these changes on experiences of policing among WWUD remain unknown. This analysis explores self-reported police interactions and pandemic-related structural vulnerability among a cohort of WWUD in Baltimore City. We used multinomial and logistic regression to explore the association of ability to meet basic needs during the pandemic with the intensity and breadth of police interaction. We observed that overlapping unmet needs, such as access to medications, bathrooms, and harm reduction supplies, were associated with exposure to more intensive enforcement and greater breadth of police practices, as well as exposure to more egregious forms of policing. Despite broad changes to both policing and social service policies amid the pandemic, our results indicate that WWUD continued to experience both disproportionately high levels of material need insecurity and exposure to police. Findings have implications for tailoring policies and interventions to meet the needs of multiply marginalized women amid big events and policy volatility.

## Introduction

The COVID-19 pandemic had an unprecedented impact on daily life. Among the most widely implemented tactics to slow transmission of the virus within communities were “stay-at-home” orders, which were gradually replaced by less restrictive social distancing policies [1]. While these orders established concrete restrictions, they largely relied on individuals assuming responsibility for managing their own risk via mask-wearing and limiting time spent in public spaces. Individuals’ ability to adopt such behaviors was influenced by a variety of structural and economic factors [2]. During the early months of the pandemic, communities with lower median incomes, greater food insecurity, and greater concentration of essential workers were found to have higher levels of activity outside of their homes [2–4]. These differences in mobility may indicate a “social distancing privilege gap [4],” in which adherence to social distancing guidelines depended on economic and material security. Prior research has demonstrated high levels of unmet material and health needs among women who use drugs (WWUD), often linked to elevated risk of psychological distress [5, 6]. Given that the COVID-19 pandemic impacted abilities to meet basic needs and exacerbated substance use-related challenges, a clearer understanding of the specific impacts among WWUD, who were already disproportionately burdened by material insecurities, is warranted.

The COVID-19 pandemic also had a notable impact on policing, with preliminary data indicating a general decline in arrest and police engagement, especially during periods of more restrictive stay-at-home orders [7, 8]. A study of 25 US cities observed that rates of drug-related crimes, property crimes, and assault significantly declined following the enactment of stay-at-home orders, while rates of shootings and homicide were not impacted [9]. While there is some evidence to suggest that the incidence of certain offenses decreased during the pandemic, there were significant changes to policing practices that might explain these trends. A study of over 1000 US law enforcement agencies found widespread reductions in in-person responses, proactive policing, and arrests in the first months of the pandemic [8]. In Baltimore, these trends were further impacted by a de facto decriminalization policy implemented by the State Attorney’s Office, in which the prosecution of low-level crime, including drug possession, was suspended to reduce incarceration [10]. However, evidence suggests that racial disparities in arrest persisted during this time. One study of arrest rates in New York City found that despite a city-wide decline in arrest in the immediate aftermath of the pandemic, the average post-pandemic arrest in Black neighborhoods remained higher than pre-pandemic arrest rates in White neighborhoods [11]. In Baltimore, post-decriminalization drug possession arrests remained disproportionately concentrated among Black individuals [12].

Outside the context of the pandemic, a strong association has been observed between material and healthcare needs and involvement with criminal-legal systems (CLS) among people who use drugs (PWUD) [13, 14]. The criminalization of drug use in the USA, including possession of illicit drugs and drug-related paraphernalia, increases PWUD’s vulnerability to arrest, incarceration, and adverse public health outcomes. PWUD’s concerns about police presence have been linked to riskier forms of drug use, including syringe sharing, rushing drug use, and using drugs in unsanitary environments [15, 16]. These behaviors are a means to evade detection, investigation, and detention by law enforcement but increase risk of infectious disease, abscess, and overdose. WWUD may be especially vulnerable to policing, due in part to their greater propensity to engage in sex work, also a criminalized behavior [17, 18]. Here, strategies to avoid police include rushing negotiations with clients, increasing risk of HIV transmission and client-perpetrated violence. The dual criminalization of drug use and sex work may also increase likelihood of police-perpetrated violence, including coercive sexual encounters with police [19].

While most research focuses on arrest and incarceration as discrete indicators of CLS involvement, encounters with law enforcement are not necessarily alike in terms of their impact on health. Lorvick et al. (2018) have proposed an “accumulation” framework that more fully accounts for the full spectrum of CLS exposure to, inclusive of police encounters besides arrest and incarceration. This includes not only the frequency and duration of singular exposures but also the breadth of this exposure across the spectrum [20]. In the context of de facto decriminalization, there is a need for increased focus on policing practices as they can occur independent from arrest. Previous research with women who exchange sex (WES) in Baltimore City pre-pandemic has demonstrated exposure to a broad range of sanctioned and unsanctioned policing practices in addition to arrest that can have significant impacts on health [21, 22]. Specifically, a distinction is drawn between enforcement practices, routine practices employed by police officers, and egregious practices, which involve an abuse of authority. One study of women who exchange sex in Baltimore City found that cisgender WES encountered an average of 1.7 egregious police practices and 2.5 enforcement practices in the previous 6 months [23].

We attempt to address this gap in the literature by exploring the association between unmet material and healthcare needs and exposure to both procedural and egregious forms of policing among WWUD during the COVID-19 pandemic. The study was conducted in the context of a period of de facto decriminalization, which would theoretically reduce police enforcement of sex work and drug-related behaviors.

## Methods

### Study Setting and Data Collection

This study utilizes data from the COVID Action Research Engagement (CARE) study of WWUD in Baltimore City, Maryland. Recruitment occurred between August 2021 and December 2022. More information about recruitment and sampling methods have been previously reported [24]. Once these areas were identified, recruitment was conducted on a mobile van where respondents were screened, consented, and enrolled in the study before completing the baseline survey (45–60 min). Eligibility criteria for study participation included self-identifying as a woman, being 18 years or older, and reporting non-medical use of drugs other than cannabis at least three times in the past 3 months. Participants were remunerated with a $50 Visa gift card and provided with harm reduction supplies, hygiene supplies, and references to local resources, if desired. All study activities, including COVID-19 safety protocols for in-person data collection, were reviewed and approved by the Johns Hopkins Bloomberg School of Public Health’s Institutional Review Board.

#### Measures

##### Dependent Variables

Exposure to policing practices was measured using an index among a population of women who exchange sex (WES) in Baltimore City [22]. This index has subsequently been used to measure police exposure among PWID in California [25]. In the present study, participants were asked whether they had encountered any of a list of 11 discrete policing practices within the past 3 months. These practices are categorized into either enforcement practices (e.g., asked for ID, confiscated drugs) or egregious practices (e.g., intimidation, destroyed property). From this index, three distinct variables were constructed to measure WWUD’s recent exposure to policing:

- Intensity of enforcement practices: The total number of enforcement practices reported by participants in the past 3 months, categorized into tertiles (0, 1, or 2+ enforcement practices).
- Any egregious practices: The total number of egregious practices reported in the past 3 months, dichotomized into any (1+) or no exposure.
- Breadth of police practices: Exposure to discrete categories of policing practices, to indicate whether participants had been exposed to *neither* enforcement nor egregious practices, *either* an enforcement or egregious practice, or *both* an enforcement and egregious practice.

##### Independent Variables

Self-reported challenges to meeting basic needs during the pandemic were measured using an adapted version of the Detroit Metro Area Communities COVID-19 Survey. For each of the 15 challenges (e.g., accessing healthcare, getting something to eat), respondents were asked whether each was not a challenge, a minor challenge, or a major challenge. Responses were dichotomized into not a challenge or either a minor or major challenge.

##### Covariates of Interest

We theorized that several variables were associated with both exposure to policing and unmet basic needs that may confound this relationship. These variables included recent sex work (binary; any vs none), recent injection drug use (binary; any versus none), and self-reported income from a criminalized behavior other than sex work (e.g., shoplifting). Given changes to local and state policy related to the COVID-19 pandemic over the study period, a variable to account for time was constructed. Observations were binned into calendar-year quarters based on the survey completion date.

#### Statistical Analysis

The association of pandemic challenges, demographic factors, and self-reported engagement in criminalized behaviors with policing practices was assessed using bivariate and multivariable regression. Multinomial logistic regression was used to examine the relative risk of experiencing one or two or more enforcement practices vs none. Logistic regression was used to examine the odds of experiencing any egregious practice vs none. To evaluate the additive impact of multiple vulnerability factors on exposure to policing, they were added in a stepwise fashion following a procedure introduced by Friedman et al. (2021) to visualize intersecting risks [25]. The effect of overlapping challenges addressing health and material needs was examined by including challenges significant at *p* < 0.10 into an adjusted model in a stepwise fashion, based on the size of coefficients in individual regression models. Linear combinations were calculated to estimate the additive effect of each pandemic challenge on exposure to policing practices.

Once the relationship between unmet needs and exposure to enforcement vs egregious practices was explored, we examined the association between unmet needs and breadth of exposure using multinomial logistic regression.

## Results

### Descriptive Statistics

The socio-demographic characteristics and self-reported encounters with criminal-legal systems among women in our sample are provided in Table 1. A majority of women in the sample reported the use of opioids (*n* = 191, 84.1%) and stimulants (199, 87.7%) in the past 3 months. Roughly a third of women reported past 3-month injection drug use (79, 34.8%). Half of the women in our sample had experienced challenges having a place to stay (108, 47.6%), getting enough food to eat (118, 52.0%), or getting where they needed to go (111, 48.9%) during the pandemic. Encounters with the criminal-legal system among women in our sample are described in Table 2. Nearly one in five women (40, 17.6%) in the sample reported that they had been arrested since March 2020. Of the 40 participants who had been arrested, 19 (47.5%) reported that they had been arrested for an offense that was explicitly covered by the State Attorney’s decriminalization policy (e.g., solicitation, loitering, drug possession). In the previous 3 months, 96 women (43%) reported that they had experienced any enforcement practice, and 25 (11%) reported any abusive practice. While 119 women (54%) reported that they had experienced neither enforcement nor egregious practices, 77 (35%) reported that they had experienced only an enforcement practice, and 20 (9%) reported they had experienced both enforcement and egregious practices in the past 3 months. Less than 2% of women in our sample reported that they had experienced an egregious practice without also experiencing any enforcement practices.

**Table 1:** Characteristics of sample of women who use drugs in Baltimore, Maryland (*n* = 227)

	Full sample (*n* = 227)
	*n* (col%)
Socio-demographics	
Age	
< 40	117 (51.5%)
40+	110 (48.5%)
Black, alone or in combination	114 (50.2%)
Education received	
< High school diploma	93 (41.0%)
High school diploma or GED	77 (33.9%)
Some college+	55 (24.2%)
Housing type	
Own housing (owns or rents)	81 (35.7%)
Shared housing (friend or family)	80 (35.2%)
Temporary housing (shelter, motel)	9 (4.0%)
Literal homelessness	51 (22.5%)
Experiences food insecurity, weekly	96 (42.3%)
Drug and alcohol use	
Any opioid use, past 3 months	191 (84.1%)
Any stimulant use, past 3 months	199 (87.7%)
Any fentanyl use, past 3 months	130 (57.3%)
Any injection drug use, past 3 months	79 (34.8%)
Sex work	
Ever sold or traded sex	143 (63.0%)
Sold or traded sex, past 3 months	120 (52.9%)
Unmet needs during COVID-19 pandemic	
Getting healthcare I need	56 (24.7%)
Getting mental healthcare I need	73 (32.2%)
Having a place to stay/live	108 (47.6%)
Getting enough food to eat	118 (52.0%)
Having clean water to drink	73 (32.2%)
Getting medicine I need	77 (33.9%)
Getting where I need to go	111 (48.9%)
Getting childcare when I need it	29 (12.8%)
Getting cleaning or paper products	90 (39.6%)
Getting PPE	62 (27.3%)
Getting condoms or safe sex supplies	51 (22.5%)
Getting naloxone	29 (12.8%)
Getting new syringes or safe use supplies	39 (17.2%)
Having a bathroom to use	82 (36.1%)
Having a place to wash up	85 (37.4%)

#### Bivariate Results

Bivariate analysis was used to explore the association between the intensity of enforcement practices or any egregious practices with socio-demographic factors and criminalized behaviors. Older women in our sample were significantly less likely to report two or more enforcement practices (relative risk ratio (RRR): 0.44, 95% confidence interval (CI): 0.20–0.99) or any egregious practice (odds ratio (OR): 0.30, 95% CI: 0.11–0.78). Women who reported selling or exchanging sex in the past 3 months were significantly more likely to report one enforcement practice (RRR: 2.33, 95% CI: 1.23–4.43) or any egregious practices (OR: 3.60, 95% CI: 1.29–10.05). Women who reported that they generated income via at least one criminalized behavior were more likely to report one enforcement practice (RRR: 2.83, 95% CI: 1.49–5.35), two or more enforcement practices (RRR: 4.51, 95% CI: 1.81–11.24), or any egregious practice (OR: 2.85, 95% CI: 1.09–7.46).

#### Intensity of Enforcement Practices

Association between pandemic-related unmet needs and intensity of enforcement practices adjusted to account for age, criminalized behaviors, and time, is displayed in Table 3. We did not observe a significant association between any pandemic-related challenge and exposure to one enforcement practice vs none. However, exposure to two or more enforcement practices was significantly associated with several pandemic-related challenges. Women were significantly more likely to have experienced two or more enforcement practices versus none if they reported challenges in having a place to live or stay (aRRR: 3.22, 95% CI: 1.27–8.19), getting needed medicine (aRRR: 2.58, 95% CI: 1.10–6.05), getting cleaning products (aRRR: 2.36, 95% CI: 1.00–5.57), or having a bathroom to use (aRRR: 3.89, 95% CI: 1.58–9.57). Women who reported challenges accessing harm reduction supplies during the pandemic were significantly more likely to experience two or more enforcement practices, including challenges getting naloxone (aRRR: 4.51, 95% CI: 1.55–13.07) or sterile syringes (aRRR: 3.88, 95% CI: 1.47–10.24).

**Table 2:** Frequency of self-reported exposure to criminal-legal encounters among women who use drugs in Baltimore City (*n* = 227)

	Full sample (*n* = 227)
	*n* (col%)
Decriminalization	
Aware of SA policy change*	125 (55.1%)
Arrest	
Arrested since March 2020	40 (17.6%)
***Arrested for:***	
Solicitation/prostitution^	8 (3.5%)
Loitering/trespassing^	4 (1.8%)
Drug/paraphernalia possession^	14 (6.2%)
Drug distribution	6 (2.6%)
Assault	9 (4.0%)
Robbery/theft	5 (2.2%)
Procedural (warrant/probation/missed court date)	7 (3.1%)
Driving violation	4 (1.8%)
Police encounters	
***Before pandemic officers said something***	
Never	130 (57.3%)
A few times a year/monthly	66 (29.7%)
Weekly/daily	26 (11.7%)
***In last year, officers said something…***	
Never	138 (60.8%)
A few times a year/monthly	62 (27.9%)
Weekly/daily	22 (9.9%)
Police practices, past 3 months	
***Enforcement practices***	
Police asked to move on	69 (30.4%)
Police asked to see ID/ran warrant	28 (12.3%)
Police conducted search	20 (8.8%)
Confiscated drugs or paraphernalia	11 (4.8%)
Referred to health or social services	15 (6.6%)
*Number of enforcement practices*	
0	127 (57.0%)
1	64 (28.7%)
*2*+	32 (14.4%)
***Egregious practices***	
Bullied/intimidated	18 (7.9%)
Touched inappropriately	5 (2.2%)
Damaged personal property	7 (3.1%)
Pressured for sex to not arrest	8 (3.5%)
Accepted money/goods to not arrest	6 (2.6%)
Physically harmed/used weapon	3 (1.3%)
*Number of egregious practices*	
0	196 (88.7%)
1	15 (6.8%)
*2*+	10 (4.5%)

* missing *n* = 45 observations; ^ arrest covered under decriminalization policy

**Fig. 1:**
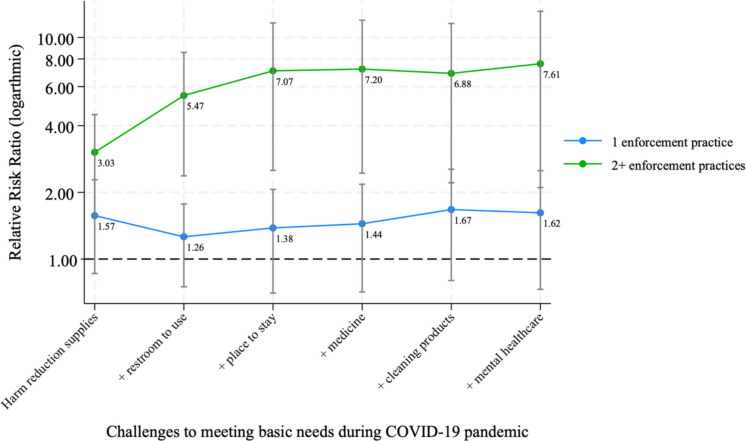
Additive effect of overlapping need challenges on the relative risk of exposure to 1 vs 2 or more enforcement practices

The additive intersectional risk of overlapping material and healthcare needs on exposure to enforcement practices is depicted in Fig. 1. The addition of overlapping unmet material and healthcare needs did not significantly increase the likelihood of experiencing a singular enforcement practice. However, experiencing multiple overlapping unmet needs significantly increased the likelihood of having experienced multiple types of enforcement practices. For example, while the likelihood of having experienced two or more enforcement practices was 5.5 times greater among women who experienced both challenges accessing harm reduction supplies and having a bathroom to use (aRRR: 5.47, 95% CI: 1.80–16.60), the likelihood was 7.1 times greater among women who additionally experienced challenges in finding a place to stay (aRRR: 7.07, 95% CI: 2.00–25.02).

### Exposure to Egregious Policing

**Fig. 2:**
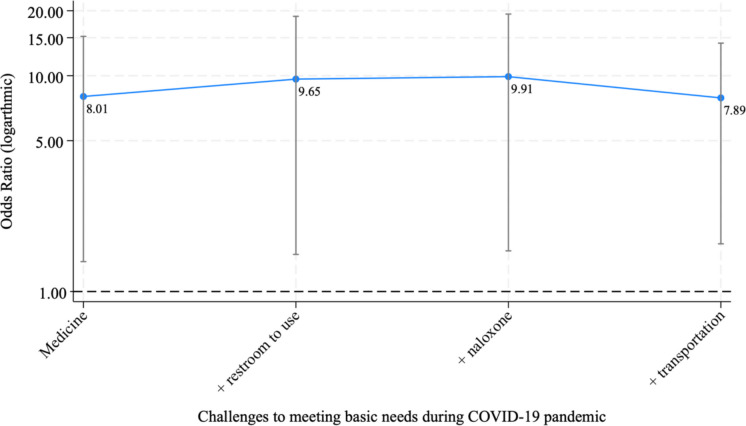
Additive effect of overlapping need challenges on the odds of exposure to egregious policing practices

Associations between pandemic-related challenges and experiencing any egregious policing practices, adjusted for age, criminalized behavior, and recruitment date, are also displayed in Table 3. Women who experienced challenges in getting medicine that they needed (aOR: 4.46, 95% CI: 1.72–11.52) or having a bathroom to use (aOR: 3.50, 95% CI: 1.34–9.17) had significantly greater odds of having experienced egregious policing. The additive intersectional risk of overlapping unmet needs on exposure to egregious practices is depicted in Fig. 2. As the quantity of unmet needs increased, so did the likelihood of having experienced any egregious policing. For example, while the odds of having experienced any egregious practice were 7.3 times greater among women who experienced challenges both getting medicine and having a bathroom to use (aOR: 7.31, 95% CI: 2.21–24.18), the likelihood was 10.4 times greater among women who also additionally experienced challenges getting naloxone (aOR: 10.36, 2.44–44.00).

#### Breadth of Policing Practices

Associations between unmet needs and exposure to either or both policing practices, adjusted for age, criminalized behavior, and recruitment date, are reported in Table 4. Women who reported challenges getting cleaning products (aRRR: 2.06, 95% CI: 1.09–3.89), or accessing needed medicines (aRRR: 1.96, 95% CI: 1.01–3.79), were significantly more likely to report having experienced either practice vs neither. Women who reported challenges getting naloxone (aRRR: 4.51, 95% CI: 1.55–13.07), finding a bathroom to use (aRRR: 6.03, 95% CI: 1.93–18.84), or accessing needed medicines (aRRR: 5.29, 95% CI: 1.70–16.44) were significantly more likely to report experiencing both categories of policing practices than neither. They were also more likely to report both practices than either practice.

**Table 3:** Adjusted regressions of pandemic challenges on exposure to enforcement or egregious policing practices

		Enforcement practice, past 3 m			Any egregious practice, past 3 m	
		One vs none	2+ vs none			
		*aRRR^^ (95% CI)*	*aRRR^^ (95% CI)*		*aOR^^ (95% CI)*	
**Pandemic challenges**						
1	Getting healthcare I need	0.67 (0.31, 1.46)	1.06 (0.42, 2.69)		1.73 (0.66, 4.51)	
2	Getting mental healthcare I need	1.17 (0.57, 2.42)	2.37 (0.97, 5.79)	*	1.99 (0.76, 5.22)	
3	Having a place to stay/live	1.00 (0.52, 1.94)	3.22 (1.27, 8.19)	**†	1.82 (0.70, 4.74)	
4	Getting enough food to eat	1.10 (0.56, 2.14)	1.26 (0.52, 3.02)		1.21 (0.46, 3.16)	
5	Having clean water to drink	1.58 (0.78, 3.20)	1.29 (0.51, 3.30)		1.55 (0.58, 4.15)	
6	Getting medicine I need	1.44 (0.73, 2.86)	2.58 (1.10, 6.05)	**	4.46 (1.72, 11.52)	**
7	Getting where I need to go	1.42 (0.74, 2.71)	1.72 (0.74, 4.01)		2.39 (0.90, 6.30)	*
8	Getting childcare when I need it	0.70 (0.25, 2.00)	1.59 (0.53, 4.74)		0.46 (0.10, 2.20)	
9	Getting cleaning or paper products	1.90 (0.97, 3.70)	2.36 (1.00, 5.57)	**	1.89 (0.75, 4.76)	
10	Getting PPE	1.33 (0.65, 2.72)	1.38 (0.55, 3.45)		1.43 (0.53, 3.84)	
11	Getting condoms or safe sex supplies	0.98 (0.44, 2.17)	2.14 (0.86, 5.30)	*†	1.85 (0.70, 4.89)	
12	Getting naloxone	1.18 (0.43, 3.24)	4.51 (1.55, 13.07)	**†	2.97 (0.98, 8.99)	*
13	Getting new syringes or safe use supplies	1.43 (0.59, 3.46)	3.88 (1.47, 10.24)	**†	2.30 (0.83, 6.35)	
14	Having a bathroom to use	1.50 (0.75, 3.02)	3.89 (1.58, 9.57)	**†	3.50 (1.34, 9.17)	**
15	Having a place to wash up	1.43 (0.72, 2.87)	2.12 (0.87, 5.14)	*	2.01 (0.78, 5.16)	

*aRRR* adjusted relative risk ratio, ^^ estimates adjusted for recruitment date, age, past 3-month injection drug use, past 3-month sex work, and generating income from criminalized behavior other than sex work; * *p* < 0.1, ** *p* < 0.05, † 2+ vs 1 enforcement practice coefficient *p* < 0.1

**Table 4:** Adjusted multinomial regression of individual pandemic challenges on breadth of exposure to categories of police practices

			Exposure to police practice categories			
			Either vs none		Both vs none	
			aRRR^^ (95% CI)		aRRR^^ (95% CI)	
**Pandemic challenges**						
1	Getting healthcare I need		1.11 (0.54, 2.27)		0.89 (0.26, 3.05)	
2	Getting mental healthcare I need		1.68 (0.86, 3.31)		1.61 (0.51, 5.10)	
3	Having a place to stay/live		1.16 (0.62, 2.18)		2.54 (0.77, 8.36)	
4	Getting enough food to eat		1.41 (0.74, 2.69)		0.92 (0.30, 2.83)	
5	Having clean water to drink		1.40 (0.71, 2.76)		1.73 (0.54, 5.50)	
6	Getting medicine I need		1.96 (1.01, 3.79)	**	5.29 (1.70, 16.44)	**†
7	Getting where I need to go		1.56 (0.85, 2.86)		2.65 (0.87, 8.06)	*
8	Getting childcare when I need it		1.22 (0.50, 3.01)		0.71 (0.13, 3.79)	
9	Getting cleaning or paper products		2.06 (1.09, 3.89)	**	2.51 (0.86, 7.37)	*
10	Getting PPE		1.35 (0.68, 2.68)		1.33 (0.40, 4.38)	
11	Getting condoms or safe sex supplies		1.12 (0.53, 2.37)		2.78 (0.88, 8.73)	*
12	Getting naloxone		1.26 (0.49, 3.26)		4.61 (1.26, 16.85)	**†
13	Getting new syringes or safe use supplies		2.14 (0.94, 4.85)	*	2.94 (0.86, 10.03)	*
14	Having a bathroom to use		1.82 (0.93, 3.56)	*	5.58 (1.74, 17.96)	**†
15	Having a place to wash up		1.65 (0.85, 3.21)		3.00 (0.98, 9.22)	*

^^ adjusted for criminalized income besides sex work, time, past 3-month sex work, past 3-month injection drug use, age, † significant difference between either vs both

## Discussion

To our knowledge, this is the first study to examine WWUD’s policing experiences amid the COVID-19 pandemic and nationwide scale back of policing. Compared to a study of WES in Baltimore City recruited using similar methods prior to decriminalization, a smaller proportion of women in our sample reported experiencing enforcement (43.6% vs 81.6%) or egregious (11.3% vs 38.0%) policing practices within the prior 3 months [22]. However, while encounters with police may have generally declined during the pandemic, findings from our study suggest that exposure to policing remained highest among women simultaneously facing unmet needs. Additionally, data illustrated a compounded burden of material insecurity with policing, as WWUD with overlapping unmet needs were more likely to face greater intensity of both police enforcement and egregious policing. While the acute phase of the COVID-19 pandemic has concluded, elaboration of WWUD’s experiences during this time can detail the amplification of risks to adverse health and social outcomes in times of social disruption, including future public health emergencies and natural disasters. This research adds to the growing body of evidence demonstrating a link between structural vulnerability and exposure to more intensive and abusive forms of policing [13, 20, 25].

Our analysis conforms with prior research demonstrating a link between socio-structural vulnerability and exposure to policing, providing evidence that this pattern persisted in the context of the COVID-19 pandemic [25]. Specifically, women struggling to face their most basic needs during the COVID-19 pandemic, including places to stay or use the bathroom, were more likely to report experiencing multiple enforcement practices. Previous studies have highlighted how unstably housed individuals were disproportionately impacted by pandemic-related changes to the delivery of health and social services, including the closure of drop-in centers and shelters [26]. Due to the closure of “safe haven” programs and substandard housing conditions, women experiencing high levels of material insecurity may have resorted to spending extended periods of time in public places while lockdown policies were in effect, likely increasing their visibility and exposure to repeated police enforcement practices. While significant investment was made to increase unstably housed individuals’ access to shelter during the pandemic, for example, the establishment of hotel-based “shelter-in-place” housing, these programs were not designed with the specific needs of PWUD in mind. Many programs enforced curfews, searched properties, and prohibited the possession or consumption of drugs on hotel premises [27, 28]. To retain PWUD in housing amid public health emergencies, there is a need to develop housing interventions that incorporate harm reduction supports.

Experiencing unmet healthcare needs during the pandemic, specifically access to needed medications, was significantly associated with exposure to more intensive and abusive forms of policing. While we did not gather information about the specific medications WWUD in our sample struggled to access, a greater proportion of women with a mental health condition or receiving MOUD treatment reported this challenge. This aligns with prior research that has demonstrated an association between greater jail frequency and unmet physical and mental health needs, highlighting both the destabilizing effect of criminal justice involvement and its contribution to avoidance of healthcare services [13]. These patterns were likely exacerbated during the pandemic, when “shelter-in-place” orders contributed to drastic changes to the delivery of healthcare services. While several initiatives attempted to promote continued access to MOUD during stay-at-home orders, such as the expansion of take-home dispensing protocols [29], research suggests that inequities in treatment access were exacerbated. This was especially true for individuals experiencing housing instability, who were unlikely to have the means to engage in telehealth and were explicitly excluded from take-home dosing [30]. Disruptions in access to needed medications, combined with heightened levels of stress caused by the pandemic, may have led to more frequent engagement with the drug market to cope, increasing vulnerability to policing.

Women who struggled to access harm reduction supplies, including sterile syringes and naloxone, were more likely to experience a greater intensity of enforcement practices, as well as different forms of policing. This suggests that while low-level drug offenses, including simple drug and paraphernalia possession, were effectively decriminalized in Baltimore City, WWUD were still disproportionately targeted by police. The role of police discretion in implementing reforms to drug enforcement has been highlighted as a possible barrier to improving criminal-legal outcomes amid recent decriminalization efforts [31]. Despite the absence of a legal requirement for police to stop arresting for crimes included in the city’s de facto policies, evidence demonstrated substantial reductions in arrests for drug and paraphernalia possession, sex work, and other adjacent crimes in Baltimore City from 2020 to 2021 [10]. However, possible impacts of the policy on other forms of police encounters, including ID checks, searches, and harassment, have not been evaluated to date. This highlights the value of an accumulation approach when studying CLS exposure, rather than focusing on singular outcomes such as arrest or incarceration. This approach accounts for the extension of policing practices into other facets of social life, including the subjection of impoverished, unhoused and participating in the street-based economy to greater surveillance and punishment [32]. Findings also highlight possible limitations of decriminalization in effecting the nature of street interactions between officers and individuals navigating multiple forms of material insecurity. As our data support, even in the absence of arrest, there is a range of routine and egregious practices that officers may employ that can lead to a cascade of negative outcomes. To effectively interrupt these cycles, there is a need for more comprehensive reform that shifts the culture and norms of policing contributing to the disproportionate targeting of PWUD by investing in resources promoting social and economic stability.

Finally, we observed an additive effect of material insecurity, intensity of enforcement practices, and exposure to egregious practices. In other words, the risk of experiencing more intensive and exploitative forms of policing incrementally increased as the quantity of unmet needs increased. The relationship between multiple, overlapping unmet needs and exposure to multiple enforcement practices had a distinct trend from exposure to a singular enforcement practice. This underscores the consistent over-policing of individuals engaged in behaviors that are considered deviant, including drug use, sex work, and homelessness. This demonstrates the inflexibility of this pattern even amid extraordinary circumstances, such as a global pandemic and a co-occurring local decriminalization of these behaviors. While routine enforcement practices, such as being asked to show ID or conducting property searches, are typically considered to be less harmful than more abusive practices, prior research has demonstrated that greater intensity of these practices has a negative impact on psychological well-being and engagement with healthcare services [33]. The significant overlap of this form of policing with material insecurity highlights the pervasive dehumanization that WWUD encounter to survive in challenging circumstances [34].

While there are several strengths to this study, there are also several limitations that should be noted. First, while we measured exposure to a variety of policing practices, we did not collect data about the intensity or frequency of this exposure. This is clearly relevant to capturing the intensity of policing and should be addressed in future studies. Additionally, significant changes to policing policies and the broader pandemic context occurred throughout the data collection period, which may have impacted our findings. While we attempted to control for time in adjusted analyses, this likely did not fully account for these time [33–35] dependent variations. Additionally, our sample was the use of targeted recruitment in seven distinct neighborhoods of Baltimore City to recruit a community-based sample of WWUD; however, our results may not be generalizable to WWUD living in other jurisdictions, especially given the implementation of a de facto decriminalization policy in Baltimore. Despite the fact that approximately half of the women in our sample were Black or African American, our analysis does not account for the role of racial discrimination, despite widely acknowledged racial disparities in criminal-legal system exposure. Future research is needed that explicitly interrogates intersections between structural vulnerability, exposure to policing, and racial minoritization. Finally, this cross-sectional analysis precludes causal inferences about the relationship between unmet needs and exposure to policing from being drawn. Future research should investigate the longitudinal relationship between socio-structural vulnerability and policing.

## Conclusion

WWUD already experience high levels of material insecurity and exclusion from safety-net health and social services programs. These unmet health and social needs are significantly associated with increased risk of exposure to a greater intensity and breadth of policing practices, as well as exposure to more egregious and abusive forms of policing. While widespread changes to both policing and the social safety net occurred in response to the enactment of COVID-19 stay-at-home orders, WWUD faced exacerbated levels of material need insecurity and persisting, disproportionately high exposure to both routine and egregious forms of policing, the effects of which may reinforce each other with consequences for their health and safety.

## Data Availability

To protect participant privacy, data for this study is not publicly available due to the sensitive nature of information gathered.
